# Abnormally Low Hemoglobin A1c Due to a Heterozygous Alpha-Globin Variant (Hb I; HBA2 c.49A>G, p.Lys17Glu) in a Geriatric Patient

**DOI:** 10.7759/cureus.116373

**Published:** 2026-09-16

**Authors:** Kourosh A Moshiri, Mahon Mahmodian

**Affiliations:** 1 Geriatrics, Eisenhower Medical Center, Rancho Mirage, USA

**Keywords:** alpha-globin variant, assay interference, diabetes mellitus, fructosamine, geriatric, hba1c, hba2, hb i, hemoglobin a1c, hemoglobinopathy

## Abstract

Hemoglobin A1c (HbA1c) is central to the screening, diagnosis, and longitudinal management of diabetes mellitus, but numerous physiologic and analytic factors can distort its accuracy. Hemoglobin variants are a well-recognized but frequently underappreciated source of spurious results, and the direction and magnitude of the error are highly method-dependent. We report an asymptomatic 75-year-old woman found to have a profoundly low HbA1c (<3.5%) in the setting of normoglycemia and a normal complete blood count. After exclusion of laboratory error and common confounders, alpha-globin gene sequencing revealed heterozygosity for the HBA2 c.49A>G (p.Lys17Glu) variant, designated Hb I. This variant has normal stability and produces no clinical phenotype in heterozygotes; its significance in this patient lies solely in its interference with HbA1c measurement. This case demonstrates that even a heterozygous, clinically silent alpha-globin variant can render HbA1c uninterpretable, and it emphasizes the particular hazard in older adults, in whom a falsely reassuring HbA1c may mask hyperglycemia or drive inappropriate treatment decisions and hypoglycemia. When HbA1c is unreliable, clinicians should rely on glucose-based diagnostic criteria and on direct glucose measurement (self-monitoring or continuous glucose monitoring), interpreting alternative biomarkers such as fructosamine with caution.

## Introduction

Since its introduction into clinical practice in the late 1970s and its adoption by the American Diabetes Association (ADA) for diabetes monitoring in the 1980s, hemoglobin A1c (HbA1c) has become the cornerstone metric for chronic glycemia, and since 2010, it has been endorsed as a diagnostic criterion for diabetes (HbA1c ≥ 6.5%) [1]. HbA1c reflects non-enzymatic glycation of the N-terminal valine of the hemoglobin beta chain and is proportional to ambient glucose over the preceding two to three months, provided erythrocyte lifespan and hemoglobin composition are normal [1].

The reliability of HbA1c is compromised whenever red-cell turnover is altered or when a hemoglobin variant interferes with the assay. Critically, both the magnitude and the direction of variant interference depend on the analytic platform: ion-exchange high-performance liquid chromatography (HPLC), capillary electrophoresis, boronate-affinity chromatography, immunoassay, and enzymatic assays each behave differently, and mass spectrometry is comparatively resistant [2-4]. Systematic method-comparison studies have documented interference from dozens of rare variants and specifically from alpha-globin variants across multiple platforms [5,6]. The National Glycohemoglobin Standardization Program (NGSP) maintains a continuously updated catalog of documented variant-by-method interferences [7]. A prior brief report linked a profoundly low HbA1c to a compound beta-globin hemoglobinopathy (homozygous hemoglobin S disease with hereditary persistence of fetal hemoglobin) in a younger primary-care patient [8]. The present report extends this teaching to a rare, clinically silent heterozygous alpha-globin variant in a geriatric patient and outlines a practical approach to glycemic assessment when HbA1c cannot be trusted.

## Case presentation

A 75-year-old woman presented to a geriatric clinic to establish care. Her history included hyperlipidemia, bilateral hip osteoarthritis, and osteopenia. Her medications were atorvastatin, vitamin D3, calcium, and topical diclofenac sodium gel. She reported no symptoms of hyperglycemia, including polyuria, polydipsia, polyphagia, blurred vision, fatigue, and unexplained weight loss; she had no history of diabetes, and no personal or family history of anemia or hemoglobinopathy. Vital signs and physical examination were unremarkable.

As part of routine diabetes screening during this initial visit, HbA1c was measured using the Bio-Rad D-100 ion-exchange HPLC method and was profoundly low (<3.5%). The laboratory noted that the result was affected by an interference with this method and suggested serum fructosamine as an alternative for monitoring glycemic control. Because of this discordant result, the patient was recalled for confirmatory and mechanistic testing. A repeat HbA1c was performed to exclude preanalytic or laboratory error, along with fasting plasma glucose, a complete blood count (CBC) with indices, iron studies, fructosamine, and vitamin E.

The repeat HbA1c was again <3.5% with the same documented assay interference. Fasting plasma glucose was 85 mg/dL, indicating normoglycemia. The CBC was essentially normal (hemoglobin 14.4 g/dL; hematocrit 46.5%; mean corpuscular volume 92.3 fL). Iron studies were normal. Together, these findings made anemia and common hematologic confounders less likely explanations for the low HbA1c and supported consideration of method-specific assay interference from a clinically silent hemoglobin variant. Fructosamine was 212 μmol/L (reference range, 205-285 μmol/L), within the normal interval and arguing against a low serum-protein glycation state, consistent with normoglycemia, and vitamin E was within normal limits, excluding vitamin E-related interference. The complete laboratory profile is summarized in Table 1.

**Table 1:** Complete laboratory profile. HbA1c: hemoglobin A1c; HPLC: high-performance liquid chromatography; MCV: mean corpuscular volume; MCH: mean corpuscular hemoglobin; RDW: red blood cell distribution width

Parameters	Patient values	Reference range
HbA1c (Bio-Rad D-100, ion-exchange HPLC)	<3.5% (assay interference flagged)	4%-6%
Fasting plasma glucose	85 mg/dL	70-105 mg/dL
WBC	8.7 x 10³/µL	3.4-10.8 x 10³/µL
RBC	5.04 M/µL	4.18-5.22 M/µL
Hemoglobin	14.4 g/dL	12.0-16.0 g/dL
Hematocrit	46.50%	34.0%-46.6%
MCV	92.3 fL	79-97 fL
MCH	28.6 pg	26.6-33.0 pg
RDW	14.40%	11.5%-14.5%
Platelet count	378.0 x 10³/µL	150-450 x 10³/µL
Fructosamine	212 µmol/L	205-285 µmol/L
Iron	113 µg/dL	50-212 µg/dL
Total iron-binding capacity (TIBC)	375 µg/dL	205-567 µg/dL
Unsaturated iron-binding capacity (UIBC)	262 µg/dL	155-355 µg/dL
Iron saturation	30.00%	20%-55%
Ferritin	54.6 ng/mL	11.0-306.8 ng/mL
Alpha-tocopherol (vitamin E)	17.4 mg/L	5.7-19.9 mg/L
Beta-gamma-tocopherol	<1.0 mg/L	<4.4 mg/L

Given a markedly low HbA1c not explained by glycemia or common confounders, alpha-globin gene sequencing was performed at a reference laboratory (Quest Diagnostics). This demonstrated heterozygosity for the c.49A>G (p.Lys17Glu) variant in the alpha 2-globin (HBA2) gene, designated Hb I. The patient was negative for deleterious missense, nonsense, small insertion and deletion, and obvious splice-site mutations in the alpha-1 gene, and negative for the −α3.7 alpha-globin deletion. Per the reference laboratory, the HBA2 c.49A>G (p.Lys17Glu) variant has normal stability, and heterozygous carriers have a normal clinical presentation; the laboratory noted that the clinical significance of the variant could not be definitively determined and recommended clinical correlation and genetic counseling. The patient remained clinically well and normoglycemic, and no hematologic intervention was required. She was counseled that HbA1c would be uninterpretable for her going forward and that future diabetes screening and monitoring would rely on direct glucose measurement, and genetic counseling was offered.

## Discussion

This case illustrates an association between a clinically silent, heterozygous alpha-globin variant and a grossly inaccurate HbA1c result on the ion-exchange HPLC assay used. Representative physiologic and analytic factors that can alter HbA1c results are summarized in Table 2. Several points merit emphasis.

**Table 2:** Selected physiologic and analytic factors that can falsely alter HbA1c results, grouped by mechanism. This table was independently created by the authors, summarizing information from multiple published sources [2-6,9]. The mechanism-based organization is the authors' own, and the table was not reproduced or adapted from any single published table. Radin informed the range of conditions included [9]. Variant-related HbA1c error is variant- and method-specific and should be verified against the National Glycohemoglobin Standardization Program (NGSP) interference database [7]. HbA1c: hemoglobin A1c

Mechanism	Direction of HbA1c error	Representative causes
Decreased erythrocyte turnover/prolonged RBC lifespan	Falsely increased	Iron-deficiency anemia; vitamin B12/folate deficiency; asplenia
Increased erythrocyte turnover/shortened RBC lifespan	Falsely decreased	Hemolytic anemia; recent blood loss; splenomegaly; recent transfusion; erythropoiesis-stimulating agents; later pregnancy
Chemically modified hemoglobin	Variable (assay-dependent)	Uremia (carbamylation); severe hyperbilirubinemia; severe hypertriglyceridemia; chronic alcohol use; chronic high-dose salicylates; lead exposure
Hemoglobin variants/abnormal hemoglobin fractions	Variant- and method-dependent (may be falsely high or low, or non-reportable)	Hb S, Hb C, Hb E, elevated Hb F, and alpha-globin variants such as Hb I
Interference with the glycation measurement	Falsely decreased	Vitamin C and vitamin E ingestion; ribavirin; interferon-alpha

Why the HbA1c was falsely low

HbA1c reflects glycated HbA as a fraction of total hemoglobin. In an individual carrying a substantial fraction of a non-HbA variant, two mechanisms can drive a spuriously low result: a genuine reduction in the pool of glycatable HbA, and-more commonly and more dramatically-analytic misidentification, in which an aberrant chromatographic peak co-elutes with or displaces the normal peaks so that the reported "HbA1c" no longer corresponds to true glycemia [2-4]. The Bio-Rad D-100 is an ion-exchange HPLC platform, the assay class most frequently affected by alpha-globin and other hemoglobin variants, and a value below 3.5% in a normoglycemic patient is best understood as an analytic artifact rather than a physiologic measure of average glucose. Older teaching that variant-related falsely low HbA1c occurs "most often with homozygous hemoglobinopathies" is an oversimplification: this heterozygous case shows that a single, clinically benign variant can invalidate the result, consistent with systematic studies of alpha-globin variants [5,6].

The analytic method matters

Because the magnitude and even direction of interference vary by platform, the specific HbA1c assay should always be identified when an implausible result is encountered, and the NGSP interference database consulted [2-4,7]. Where clinically important, confirmation on an orthogonal method (e.g., boronate-affinity chromatography or liquid chromatography-tandem mass spectrometry (LC-MS/MS), which are less susceptible to many variants) clarifies whether the abnormality is analytic. Recognizing the variant also spares the patient repeated, unnecessary evaluation of future spurious results.

Novelty

Interference by rare and alpha-globin variants with HbA1c is well established [5,6]. The contribution here is the identification of a specific, clinically silent heterozygous HBA2 variant (c.49A>G, p.Lys17Glu; Hb I) in association with a profoundly low HbA1c result in a geriatric patient. To our knowledge, case-level reports specifically attributing an abnormally low HbA1c to the heterozygous Hb I variant are rarely described in the clinical literature. Because the reference laboratory could not definitively establish the clinical significance of this variant, the association reported here is based on exclusion of alternative causes and the known behavior of alpha-globin variants in HbA1c assays.

Particular relevance to geriatric care

An unrecognized falsely low HbA1c is not a mere laboratory curiosity in older adults. It can create false reassurance, mask clinically significant hyperglycemia, or-if misread as "excellent control" or overtreatment-drive inappropriate escalation or de-escalation of glucose-lowering therapy. Glucose-lowering agents account for a large share of emergency hospitalizations for adverse drug events in older adults, almost all for hypoglycemia; overtreatment to low HbA1c targets is common; and the American Geriatrics Society Choosing Wisely initiative advises against using medications other than metformin to achieve HbA1c < 7.5% in most older adults [10-12]. A valid measure of glycemia is therefore essential to individualized, safety-oriented geriatric diabetes care. At the same time, investigation of an incidental laboratory abnormality in an older adult should be proportionate and aligned with the patient’s goals, comfort, and dignity. The identification of this variant at age 75 reflects its clinically silent nature and the absence of any previous clinical indication for testing; it became apparent only through method-specific HbA1c assay interference. This case should not be interpreted as supporting routine genetic testing or exhaustive investigation of every discordant HbA1c result. Here, the limited purpose of the targeted evaluation was to determine whether HbA1c could be used reliably in future care and to prevent repeated investigations, misclassification of glycemic status, and inappropriate treatment. Clinicians should individualize such evaluations and pursue additional testing only when the anticipated clinical benefit justifies the burden.

Approach when HbA1c is unreliable

Diagnosis should rely on ADA glucose-based criteria: fasting plasma glucose ≥ 126 mg/dL, two-hour plasma glucose ≥ 200 mg/dL during a 75 g oral glucose tolerance test, or random plasma glucose ≥ 200 mg/dL with classic hyperglycemic symptoms; the ADA specifically recommends plasma-glucose criteria for individuals with interfering hemoglobin variants [1]. For monitoring, structured self-monitoring of blood glucose or continuous glucose monitoring (CGM) provides direct assessment. Alternative biomarkers such as fructosamine and glycated albumin are ADA-recognized options when HbA1c is unreliable but carry a weaker evidence base; recent CGM-referenced data indicate they perform comparably to-but not clearly better than-HbA1c and do not fully overcome the limitations prompting their use [13,14]. Accordingly, direct glucose measurement-rather than fructosamine alone-is the preferred basis for this patient's future glycemic assessment. A limitation of this report is that HbA1c was not confirmed on an orthogonal, variant-resistant method (e.g., boronate-affinity chromatography or LC-MS/MS); the analytic nature of the low result is instead inferred from the flagged assay interference, normoglycemia, normal hematologic and iron indices, and the identified alpha-globin variant.

## Conclusions

An abnormally low HbA1c is not diagnostic of a hemoglobinopathy; definitive identification requires dedicated hemoglobin analysis or genetic testing. An unexpectedly low HbA1c should prompt structured evaluation: confirm the result, correlate with direct glucose measurement, exclude anemia and other turnover-related confounders, and pursue hemoglobinopathy/alpha-globin testing when the value remains discordant. The heterozygous HBA2 c.49A>G (p.Lys17Glu) variant (Hb I)-clinically silent, with normal stability and normal hematologic parameters-was associated in this patient with a profoundly low, uninterpretable HbA1c. Because variant interference is method-specific, clinicians should identify the assay in use and consult the NGSP interference resource. In geriatric patients especially, recognizing this pitfall prevents falsely reassuring interpretations and guides clinicians toward glucose-based diagnosis and monitoring.

## References

[1] Kalyani RR, Neumiller JJ, Maruthur NM, Wexler DJ (2025). Diagnosis and treatment of type 2 diabetes in adults: a review. JAMA.

[2] Zechmeister B, Erden T, Kreutzig B (2022). Analytical interference of 33 different hemoglobin variants on HbA1c measurements comparing high-performance liquid chromatography with whole blood enzymatic assay: a multi-center study. Clin Chim Acta.

[3] Yadav N, Kumar Mandal A (2023). Interference of hemoglobin variants in HbA(1c) quantification. Clin Chim Acta.

[4] Song Y, Xu A, Wang M, Shi J, Fu W, Ji L, Zhang R (2024). Evaluation of effects from hemoglobin variants on HbA(1c) measurements by different methods. Clin Chem Lab Med.

[5] Little RR, La'ulu SL, Hanson SE, Rohlfing CL, Schmidt RL (2015). Effects of 49 different rare Hb variants on HbA1c measurement in eight methods. J Diabetes Sci Technol.

[6] Xu A, Chen W, Xia Y, Zhou Y, Ji L (2018). Effects of common hemoglobin variants on HbA1c measurements in China: results for α- and β-globin variants measured by six methods. Clin Chem Lab Med.

[7] (2026). NGSP. HbA1c assay interferences. https://ngsp.org/interf.asp.

[8] St Louis J, Valdini A (2019). Abnormally low hemoglobin A1c as harbinger of hemoglobinopathy. J Am Board Fam Med.

[9] Radin MS (2014). Pitfalls in hemoglobin A1c measurement: when results may be misleading. J Gen Intern Med.

[10] Lipska KJ, Ross JS, Miao Y, Shah ND, Lee SJ, Steinman MA (2015). Potential overtreatment of diabetes mellitus in older adults with tight glycemic control. JAMA Intern Med.

[11] AGS Choosing Wisely Workgroup (2013). American Geriatrics Society identifies five things that healthcare providers and patients should question. J Am Geriatr Soc.

[12] McCoy RG, Lipska KJ, Yao X, Ross JS, Montori VM, Shah ND (2016). Intensive treatment and severe hypoglycemia among adults with type 2 diabetes. JAMA Intern Med.

[13] Daya NR, Fang M, Shin JI (2025). Detecting hyperglycemia using biomarkers versus continuous glucose monitoring. Diabetes Care.

[14] American Diabetes Association Professional Practice Committee for Diabetes (2026). 6. Glycemic goals, hypoglycemia, and hyperglycemic crises: standards of care in diabetes-2026. Diabetes Care.

